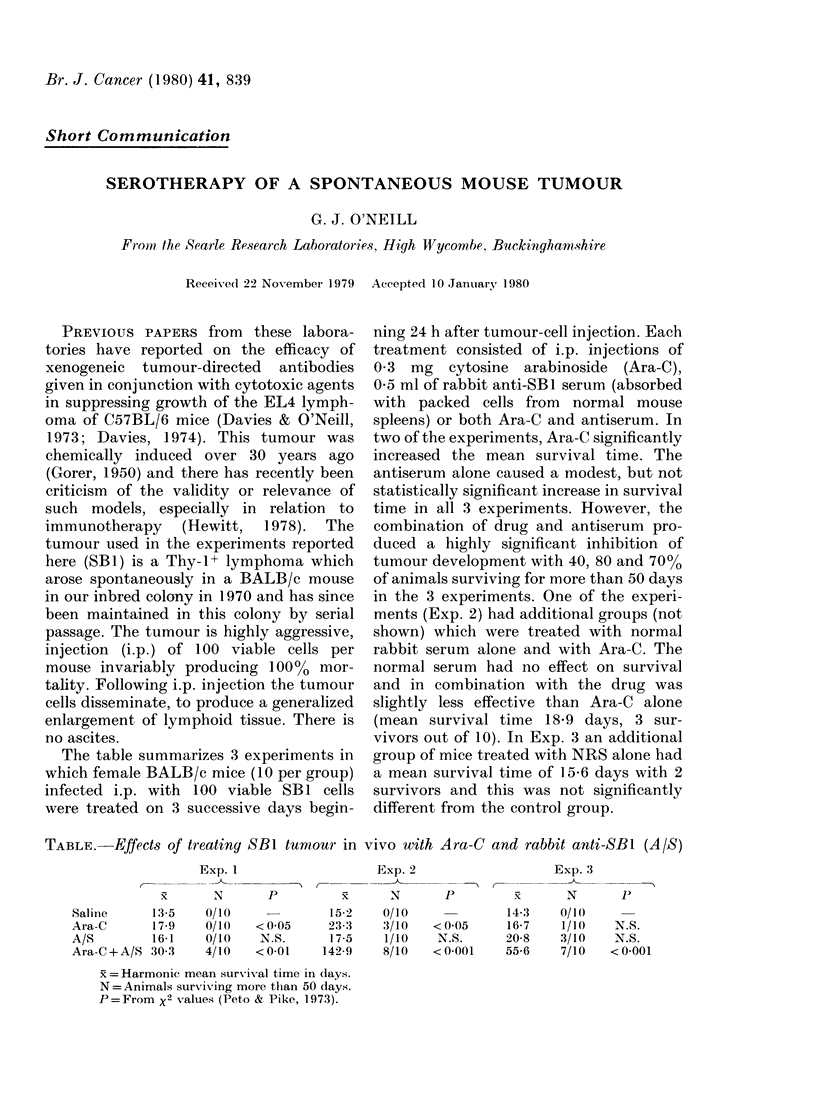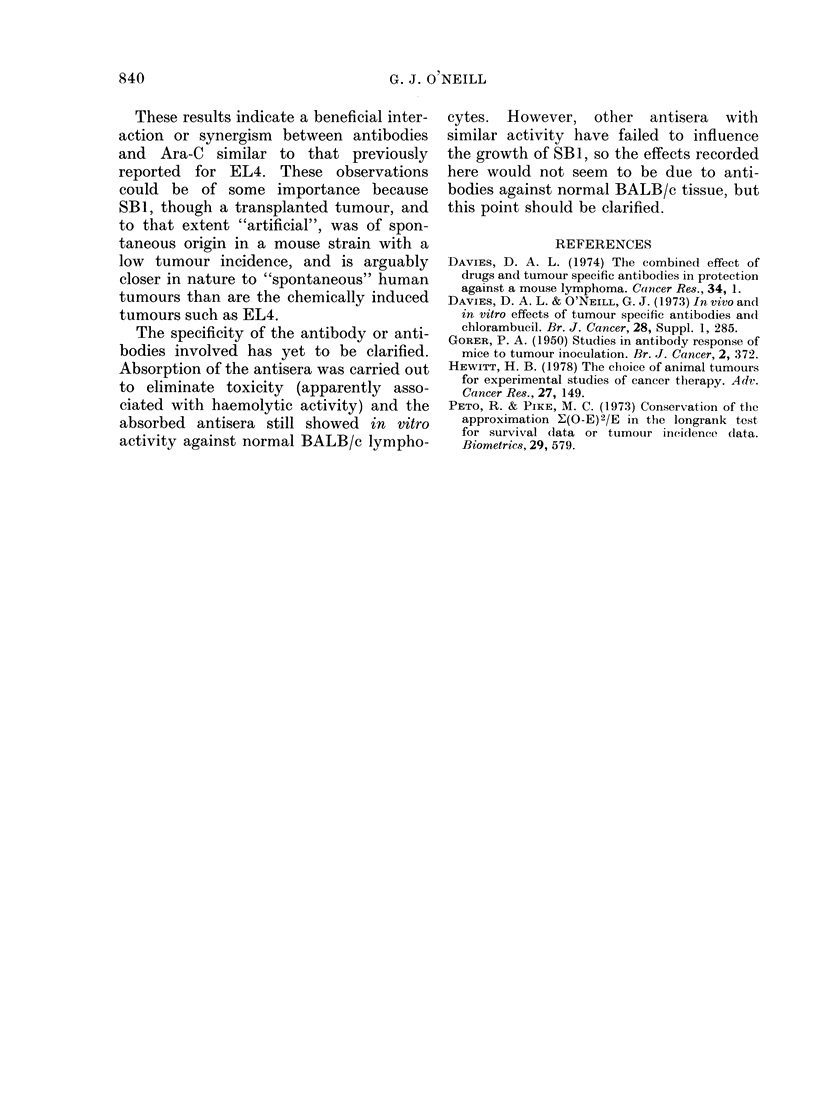# Serotherapy of a spontaneous mouse tumour.

**DOI:** 10.1038/bjc.1980.152

**Published:** 1980-05

**Authors:** G. J. O'Neill


*Br. J. Cancer* (1980) **41**, 839

***Short Communication***

### SEROTHERAPY OF A SPONTANEOUS MOUSE TUMOUR

G. J. O'NEILL

*From the Searle Research Laboratories, High Wycombe, Buckinghamshire*

Received 22 November 1979 Accepted 10 January 1980

PREVIOUS PAPERS from these laboratories have reported on the efficacy of xenogeneic tumour-directed antibodies given in conjunction with cytotoxic agents in suppressing growth of the EL4 lymphoma of C57BL/6 mice (Davies & O'Neill, 1973; Davies, 1974). This tumour was chemically induced over 30 years ago (Gorer, 1950) and there has recently been criticism of the validity or relevance of such models, especially in relation to immunotherapy (Hewitt, 1978). The tumour used in the experiments reported here (SB1) is a Thy-$1^+$ lymphoma which arose spontaneously in a BALB/c mouse in our inbred colony in 1970 and has since been maintained in this colony by serial passage. The tumour is highly aggressive, injection (i.p.) of 100 viable cells per mouse invariably producing 100% mortality. Following i.p. injection the tumour cells disseminate, to produce a generalized enlargement of lymphoid tissue. There is no ascites.

The table summarizes 3 experiments in which female BALB/c mice (10 per group) infected i.p. with 100 viable SB1 cells were treated on 3 successive days beginning 24 h after tumour-cell injection. Each treatment consisted of i.p. injections of 0·3 mg cytosine arabinoside (Ara-C), 0·5 ml of rabbit anti-SB1 serum (absorbed with packed cells from normal mouse spleens) or both Ara-C and antiserum. In two of the experiments, Ara-C significantly increased the mean survival time. The antiserum alone caused a modest, but not statistically significant increase in survival time in all 3 experiments. However, the combination of drug and antiserum produced a highly significant inhibition of tumour development with 40, 80 and 70% of animals surviving for more than 50 days in the 3 experiments. One of the experiments (Exp. 2) had additional groups (not shown) which were treated with normal rabbit serum alone and with Ara-C. The normal serum had no effect on survival and in combination with the drug was slightly less effective than Ara-C alone (mean survival time 18·9 days, 3 survivors out of 10). In Exp. 3 an additional group of mice treated with NRS alone had a mean survival time of 15·6 days with 2 survivors and this was not significantly different from the control group.

TABLE.—*Effects of treating SB*1 *tumour* in vivo *with Ara-C and rabbit anti-SB*1 (*A/S*)

| | Exp. 1 | | | Exp. 2 | | | Exp. 3 | | |
|---|---|---|---|---|---|---|---|---|---|
| | x̄ | N | *P* | x̄ | N | *P* | x̄ | N | *P* |
| Saline | 13·5 | 0/10 | — | 15·2 | 0/10 | — | 14·3 | 0/10 | — |
| Ara-C | 17·9 | 0/10 | <0·05 | 23·3 | 3/10 | <0·05 | 16·7 | 1/10 | N.S. |
| A/S | 16·1 | 0/10 | N.S. | 17·5 | 1/10 | N.S. | 20·8 | 3/10 | N.S. |
| Ara-C+A/S | 30·3 | 4/10 | <0·01 | 142·9 | 8/10 | <0·001 | 55·6 | 7/10 | <0·001 |

x̄ = Harmonic mean survival time in days.
N = Animals surviving more than 50 days.
*P* = From $\chi^2$ values (Peto & Pike, 1973).

These results indicate a beneficial interaction or synergism between antibodies and Ara-C similar to that previously reported for EL4. These observations could be of some importance because SB1, though a transplanted tumour, and to that extent "artificial", was of spontaneous origin in a mouse strain with a low tumour incidence, and is arguably closer in nature to "spontaneous" human tumours than are the chemically induced tumours such as EL4.

The specificity of the antibody or antibodies involved has yet to be clarified. Absorption of the antisera was carried out to eliminate toxicity (apparently associated with haemolytic activity) and the absorbed antisera still showed *in vitro* activity against normal BALB/c lymphocytes. However, other antisera with similar activity have failed to influence the growth of SB1, so the effects recorded here would not seem to be due to antibodies against normal BALB/c tissue, but this point should be clarified.

### REFERENCES

DAVIES, D. A. L. (1974) The combined effect of drugs and tumour specific antibodies in protection against a mouse lymphoma. *Cancer Res.*, **34,** 1.

DAVIES, D. A. L. & O'NEILL, G. J. (1973) *In vivo* and *in vitro* effects of tumour specific antibodies and chlorambucil. *Br. J. Cancer*, **28,** Suppl. 1, 285.

GORER, P. A. (1950) Studies in antibody response of mice to tumour inoculation. *Br. J. Cancer*, **2,** 372.

HEWITT, H. B. (1978) The choice of animal tumours for experimental studies of cancer therapy. *Adv. Cancer Res.*, **27,** 149.

PETO, R. & PIKE, M. C. (1973) Conservation of the approximation $\Sigma(O\text{-}E)^2/E$ in the longrank test for survival data or tumour incidence data. *Biometrics*, **29,** 579.